# Comparative Metabolomic and Pharmacognostic Analysis of *Ocimum basilicum* L. Extracts: Insights Into Antioxidant, Toxicity, and Anticancer Potentials

**DOI:** 10.1155/ijfo/1819384

**Published:** 2025-12-05

**Authors:** Vaibhavi Srivastava, Ann Maria Joseph, Priyesh Kumar, Khairah Ansari, Krupali Trivedi, Nilam Parmar, Bhakti Patel, Ashish Patel, Dipak Kumar Sahoo, Devendrasinh Jhala

**Affiliations:** ^1^ Department of Zoology, Biomedical Technology, Human Genetics and Wildlife Biology & Conservation, University School of Sciences, Gujarat University, Ahmedabad, Gujarat, India, gujaratuniversity.org.in; ^2^ Department of Life Sciences, University School of Sciences, Gujarat University, Ahmedabad, Gujarat, India, gujaratuniversity.org.in; ^3^ Department of Life Sciences, Hemchandracharya North Gujarat University, Patan, Gujarat, India, ngu.ac.in; ^4^ Department of Veterinary Clinical Sciences, College of Veterinary Medicine, Iowa State University, Ames, Iowa, USA, iastate.edu

**Keywords:** anticancer, antioxidant, GC-MS, in vitro toxicity, *Ocimum basilicum* L.

## Abstract

**Purpose:**

*Ocimum basilicum* L., a medicinal herb from the Lamiaceae family, has been traditionally used to treat various diseases. Plant extracts are valued in therapeutic medicine for their rich bioactive compounds. Selecting the appropriate extraction method and solvent is crucial for obtaining superior‐grade plant extracts. This research is aimed at characterizing the phytochemical profiles and potential biological activities of hydromethanolic (HME) and *n*‐hexane (HE) extracts derived from *Ocimum basilicum* L. leaves using the soxhlation extraction method.

**Methods:**

Extracts were subjected to physiochemical analysis (FTIR, GC‐MS, and TLC/HPTLC), quantitative analysis (total phenolic, flavonoid, and tannin content), antioxidant activity (DPPH, ABTS, NO, and SO assays), toxicity (brine shrimp and hemolysis), and anticancer activity (MTT).

**Results:**

FTIR analysis revealed major functional groups in HME and fewer in HE. GC‐MS analysis identified various compounds in HME, including estragole, beta‐farnesene, alpha‐bergamotene, alpha‐copaene, eicosapentaenoic acid, and neophytadiene, while HE contained estragole, 1‐heptatriacotanol, alpha‐bergamotene, and tau‐cadinol. TLC/HPTLC analysis reflects that both extracts contain complex mixtures of bioactive compounds. HME exhibited significantly higher levels of phenolics (122.72 ± 4.02 mg GAE/g), flavonoids (42.47 ± 0.59 mg QE/g), and tannins (30.91 ± 0.31 mg TAE/g) compared to HE (75.94 ± 0.93, 26.65 ± 1.17, and 1.99 ± 0.07 mg/g, respectively). In addition, HME exhibited significantly stronger antioxidant activity, as evidenced by lower IC_50_ values across all assays (DPPH, ABTS, NO, and SO) compared to HE. Both HME and HE exhibited notable cytotoxic activity against HCT 116, MCF‐7, and HeLa cancer cell lines in MTT assays. HE showed significantly greater cytotoxicity than HME at both 24 and 48 h. However, HME showed no toxicity in the brine shrimp lethality assay even at 2000 *μ*g/mL and exhibited no hemolytic activity, highlighting its strong biocompatibility. Conversely, HE presented mild toxicity (LC_50_ = 992 ± 88.34 *μ*g/mL) and significant hemolytic effects, indicating potential risks to erythrocytes, limiting its therapeutic use unless mitigated.

**Conclusion:**

Integrating metabolomics and pharmacognostic studies provided a comprehensive understanding of *Ocimum basilicum* L. extracts, highlighting their quality, safety, and medicinal potential.

## 1. Introduction

Among the many plants recognized for their pharmacological potential, sweet basil (*Ocimum basilicum* L.) stands out as an Ayurvedic herb traditionally used to treat conditions such as headaches, coughs, constipation, warts, worms, and kidney disorders [1]. Belonging to the Lamiaceae family, it is also valued for its therapeutic significance like antiviral, antibacterial, and antimicrobial [2], antifungal [3], cytoprotective [4], antioxidant [5], hypoglycemic [6], hepatoprotective [7], neuroprotective [8], and anticancer [9].

The present study is aimed at exploring the pharmacological advantages of *Ocimum basilicum* L. leaf extract using insights from both modern and traditional approaches by identifying the phytochemical profile of its hydromethanolic (HME) and *n*‐hexane (HE) extracts and examining their potential biological effects as chemopreventive or chemotherapeutic options for cancer management. The findings could bridge a significant gap in existing literature by providing a comparative analysis of the phytochemical and biological properties of basil leaf extracts. This could pave the way for further research and potentially lead to the development of novel cancer treatment strategies derived from traditional herbal medicine.

## 2. Materials and Methods

### 2.1. Collection of *Ocimum basilicum* L. Leaves

Fresh leaves of *Ocimum basilicum* L. were collected from Ahmedabad (23.0387° N, 72.5445° E) in the month of October 2022.

### 2.2. Authentication of *Ocimum basilicum* L.

The plant used in the study was authenticated (*Ocimum basilicum* L.) by a taxonomist. Plant samples were validated at Gujarat Biotechnology Research Centre (GBRC) via DNA barcoding. Leaf samples were lysed with PrepMan Ultra buffer (Thermo Fisher), and Internal Transcribed Spacer (ITS) primers were used with Takara EmeraldAmp GT PCR Master Mix for amplification. Sequencing employed Thermo Fisher BigDye Terminator V3.1 and an Applied Biosystems 3500xL Genetic Analyzer. Electropherograms were analyzed using CodonCode Aligner and compared with BLASTn (nr/nt) for species confirmation.

### 2.3. Preparation of *Ocimum basilicum* L. Leaves Extract

The leaves were meticulously cleaned, shade‐dried at room temperature, powdered, and stored in an airtight container for future use [10].

The Soxhlet extraction technique was employed to obtain HME and HE extracts from 5 g of powdered *Ocimum basilicum* L. leaves. HME was extracted using a methanol–water mixture (7:3) at 60°C for 9 h, targeting polar and semipolar phytochemicals. HE was prepared using 200 mL of HE at 65°C for 6 h to extract nonpolar compounds. Analytical‐grade solvents (purity ≥ 99%) ensured reproducibility and extraction efficiency. The extracts were filtered through Whatman filter paper No. 1, and solvents were removed by evaporation at 37°C–40°C. Final extracts were stored at 4°C for subsequent metabolomic and pharmacognostic investigations.

### 2.4. Determination of pH and Percentage Yield

The pH of HME and HE was assessed using the instrument Cole‐Parmer pH 5 pH Tester.

Percentage yield of HME and HE was calculated by the following formula:

Percentage yield %=WtW0×100

where *W*_t_ = weight of product after evaporation of solvent (grams) and *W*_0_ = weight of powder used in Soxhlet system (grams).

### 2.5. Physicochemical Analysis

#### 2.5.1. Fourier Transform Infrared (FTIR) Analysis

Approximately 2 mg of the extracts was placed on the ATR crystal and pressed down using the swivel press to ensure optimal contact between the sample and the crystal. The results were recorded using a BRUKER Alpha Platinum ATR FTIR spectrometer, within the range of 500–4000 cm^−1^ and with a resolution of 8 cm^−1^. The analysis of results was conducted utilizing OPUS V7.5 software.

#### 2.5.2. GC‐MS (Gas Chromatography–Mass Spectrometry) Analysis

HME and HE were analyzed using a Clarus 680 GC coupled with a Clarus SQ 8 C mass spectrometer and TurboMatrix 16 headspace sampler (PerkinElmer), following Carro et al. [11]. The GC utilized an Elite‐5 ms capillary column (30 m × 0.25 mm I.D., 0.25 *μ*m) with a 5% diphenyl/95% dimethylpolysiloxane stationary phase. Data analysis was performed using TurboMass GC/MS software and compared with the NIST 14 library. Helium was the carrier gas at 1 mL/min flow, with a 3‐min solvent delay. The oven program began at 50°C (1‐min hold), ramped at 7°C/min to 180°C (no hold), then at 50°C/min to 280°C (10‐min hold). The injection temperature was 280°C with a 10:1 split ratio. Transfer and source temperatures were maintained at 280°C. Scanning was conducted over 50–600 Da using a 30.0 m × 250 *μ*m column.

### 2.6. Thin Layer Chromatography (TLC) and High‐Performance Thin Layer Chromatography (HPTLC)

For TLC analysis, silica gel 60F254 TLC plate (6 × 2 cm) (Sigma‐Aldrich, Germany) was spotted with 10 *μ*L of HME and HE. The extracts (1 mg/mL) were prepared in dichloromethane (DCM) and filtered through a 0.22‐*μ*m syringe filter, while HPTLC analysis was carried out using a 10 × 10 cm silica gel 60F254 TLC plate on a semiautomated CAMAG Linomat 5 applicator. Using a Hamilton microsyringe (Switzerland) controlled by visionCATS software Version 2.5, a sample of 8 mm width was applied using Linomat 5 applicator under a nitrogen flow of 150 nL/s. Plates were developed in toluene:acetone:formic acid (2.25:0.375:0.375, v/v) solvent system. The plate was treated with a fresh 1% vanillin/sulfuric acid reagent, followed by heating at 110°C for a minute to visualize the bands.

### 2.7. Determination of Total Phenolic, Flavonoid, and Tannin Contents

The total phenolic [12], flavonoid [13], and tannin [14] contents in *Ocimum basilicum* L. leaf extracts were estimated. The concentration of phenolics, flavonoids, and tannins was detected with the help of a regression formula prepared using the graph of concentrations of gallic acid, quercetin, and tannic acid, respectively. The standards were employed at concentrations of 10–50 *μ*g/mL, while the sample concentration was 1 mg/mL. The absorbance was measured utilizing the BioTek Epoch Microplate Spectrophotometer (Agilent Instruments, United States), equipped with Gen5 software (Version 3.04). Each experiment was conducted in triplicates.

### 2.8. In Vitro Antioxidant Activity

In vitro free radical scavenging activity of HME and HE was examined using a spectroscopic method against DPPH^•^ (2,2‐diphenyl‐1‐picrylhydrazyl) [15], ABTS^•^ (3‐ethylbenzothiazoline‐6‐sulfonic acid) diammonium salt [16], superoxide (SO) [17], and nitric oxide (NO) [18]. Ascorbic acid (200–1000 *μ*g/mL) was used as the standard. Each experiment was performed in triplicates. The absorbance was measured utilizing the BioTek Epoch Microplate Spectrophotometer (Agilent Instruments, United States), equipped with Gen5 software (Version 3.04). IC_50_ was calculated by linear regression analysis of dose–response curve plotting between percentage scavenging activity and concentration using GraphPad Prism (V9.4.0). Each experiment was conducted in triplicates.

The antioxidant activity was calculated according to the following formula:

 Percentage antioxidant activity %=Ab−AsAb×100

where *A*_b_ = absorbance of blank and *A*_s_ = absorbance of sample.

### 2.9. MTT (3‐(4,5‐Dimethylthiazol‐2‐yl)‐2,5‐Diphenyltetrazolium Bromide) Assay

HCT 116 (human colorectal cancer), MCF‐7 (human breast cancer), and HeLa (human cervical cancer) cell lines were obtained from NCCS, Pune, India. HCT 116 cells were cultured in DMEM, while MCF‐7 and HeLa cells were maintained in RPMI 1640 media, both supplemented with 10% FBS, penicillin (100 U/mL), and streptomycin (100 *μ*g/mL). Cells were incubated at 37°C in a humidified atmosphere with 5% CO_2_.

The cytotoxic effects of HME and HE extracts were assessed via the MTT assay [19]. Cells (1 × 10^5^/well) were seeded in 96‐well plates with 200 *μ*L medium and examined under a ZEISS Primovert inverted microscope at 20× magnification. Extracts were dissolved in sterile distilled water, filtered (0.22 *μ*m), and tested at concentrations of 2–1024 *μ*g/mL. Untreated cells served as controls. After 24‐ and 48‐h exposures, media were aspirated, wells were washed with PBS, and 30 *μ*L of MTT solution (5 mg/mL) was added for 4 h at 37°C. Formazan crystals were dissolved in 100 *μ*L DMSO, and absorbance was measured at 570 nm using a BioTek Epoch Spectrophotometer. IC_50_ values were determined graphically, with experiments performed in triplicate.

Percentage cell viability was calculated using the formula below:

Percentage cell viability %=AtAc×100

where *A*_t_ = absorbance value of treated cells and *A*_c_ = absorbance value of control.

### 2.10. Toxicity Testing

#### 2.10.1. Brine Shrimp Lethality Assay (BSLA)

The BSLA was conducted following the protocol outlined by Meyer and coworkers in 1982 [20]. About 5 mg brine shrimp (*Artemia salina*) eggs are allowed to hatch after 24 h of incubation, developing into first stage larvae. The 10 nauplii were exposed to varying concentrations (200, 400, 600, 800, 1000, 1500, and 2000 *μ*g/mL) of the HME and HE. Potassium dichromate, K_2_Cr_2_O_7_ (10, 20, 30, 40, and 50 ppm), was taken as a test control. For the negative control, 2 mL of artificial sea water was added instead of the sample. After 24 h, live nauplii were counted, and the percentage mortality was determined. Each experiment was performed in triplicates.

#### 2.10.2. Hemolysis Assay

The hemolytic activity of HME and HE was evaluated using human erythrocytes by following the method described by Gaonkar and coworkers in 2017 [21]. Freshly collected blood (5 mL) in a heparinized vacutainer was centrifuged (1500 rpm, 5 min), and the pellet was washed thrice with PBS (pH 7.2 ± 0.2). A 2% erythrocyte suspension was prepared, and 500 *μ*L aliquots were treated with 25 *μ*L of extracts (8–1024 *μ*g/mL), positive control (10% Triton X‐100), or negative control (PBS). Samples were incubated at 37°C for 1 h, centrifuged (1500 rpm, 10 min), and the absorbance of supernatant was measured at 570 nm to calculate the percentage hemolytic activity using the following formula:

Percentage hemolytic activity %=As−AncApc−Anc×100

where *A*_s_ = absorbance of sample, *A*_nc_ = absorbance of negative control (PBS), and *A*_pc_ = absorbance of positive control (Triton X‐100).

### 2.11. Statistical Analysis

Statistical analysis of the data was conducted using GraphPad Prism (Version 9.4.0), and results were presented as mean ± standard error (SE). Statistical significance was determined as follows:  ^∗^ *p* < 0.05,  ^∗∗^ *p* < 0.01,  ^∗∗∗^ *p* < 0.001, and^∗∗∗∗^ *p* < 0.0001.

## 3. Results

### 3.1. Authentication of *Ocimum basilicum* L. Through DNA Barcoding

The plant sample was verified as *Ocimum basilicum* L. (Herbarium No. GU/BOT/L1) through detailed morphological examination by a principal taxonomist. Further, the plant was validated using DNA barcoding. Obtained *Ocimum basilicum* L. nucleotide sequences were queried for highly similar sequences from NCBI using the nucleotide BLAST tool in NCBI GenBank.

Following is the nucleotide sequence obtained from the ITS region of *Ocimum basilicum* L.:

GGGGTCGCGGTCGAGGTACTTGGGTTTTTAAAGGGGATCCGCACGGACGACCCGGGGCGCGACGGGGAATTTGATAAGTTCAACCACCACTTGTCGCGACGCGGGTCGCCGGGGGATCGCATTTGGGCCGGCCGCGCAACGGGGGCCCGGGAGGCCATTTTCCGCCCCCCCCAAGCCCAACGCATTGCGGGGGGATGGGGGGGCGACGCATGCGTGACGCCCAGGCAGACGTGCCCTCGGCCTGAT.

The results of DNA barcoding indicate a strong match between the sequenced region and the reference sequence of *Ocimum basilicum* L. in the NCBI database (Table 1).

**Table 1:** Result of BLASTn against NCBI standard database.

**Sample ID**	**Identified species**	**Query length (bp)**	**Max score**	**Query cover**	**E** **-value**	**Per. Ident.**	**Acc. Len.**	**Subject accession**
Ocimum	*Ocimum basilicum*	247	364	100%	3.00e − 102	93.12%	432	MZ190983.1

Abbreviations: Acc. Len., accession length; Per. Ident., percent identity.

### 3.2. Determination of pH and Percentage Yield

HME and HE differ significantly in both pH and percentage yield. HME was found to be mildly acidic (pH 6.8) with a higher percentage yield (12.20%), while HE was neutral (pH 7) with a lower percentage yield (3.69%). These differences could stem from variations in their chemical compositions, reaction conditions, or extraction methods used to obtain these substances.

### 3.3. Physiochemical Analysis

#### 3.3.1. FTIR Analysis

The functional groups present in the HME and HE extracts of *Ocimum basilicum* L. were identified using FTIR spectroscopy by analyzing their infrared absorption spectra. Figure 1 presents the FTIR spectra of HME (a) and HE (b), while Table 2 provides the corresponding functional group assignments for both extracts.

Figure 1FTIR spectrum of (a) HME and (b) HE.

**Figure:**
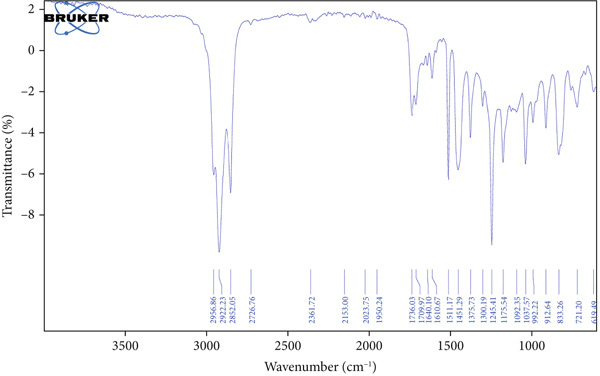
(a)

**Figure:**
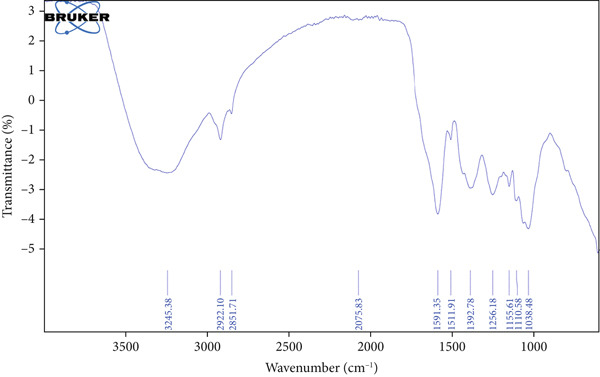
(b)

**Table 2:** FTIR frequency range, characteristic bond and functional groups of HME and HE.

**Absorption frequency (cm** ^**−1**^ **)**	**Characteristic bond**	**Functional group**	**HME**	**HE**
3550–3200; 3200–2700	O‐H stretching	Alcohol	+	+
2140–1990	N=C=S stretching	Isothiocyanate	+	−
2000–1900	C=C=C stretching	Allene	+	−
1750–1735; 1210–1163	C=C stretching	Esters	+	−
1720–1706	C=O stretching	Carboxylic acid	+	−
1690–1640	C=N stretching	Imine/oxime	+	−
1620–1610	C=C stretching	*α*,*β*‐unsaturated ketone	+	−
1550–1500	N‐O stretching	Nitro compound	+	+
1450	C‐H stretching	Alkane	+	−
1400–1000	C‐F stretching	Fluoro compound	+	+
1390–1310	O‐H stretching	Phenol	+	−
1350–1300	S=O stretching	Sulfone	+	−
1250–1020	C‐N stretching	Amine	+	−
1275–1200	C‐O stretching	Alkyl aryl ether	−	+
1124–1087	C‐O stretching	Secondary alcohol	+	−
1070–1030	S=O stretching	Sulfoxide	+	−
995–985; 840–790; 730–665	C=C stretching	Alkene	+	−
600–500	C‐Br stretching	Halo compound	+	−

*Note:* + present, − absent.

#### 3.3.2. GC‐MS Analysis

The bioactive compounds in HE and HME were identified using GC‐MS. The results, detailed in Table 3 and illustrated in Figure 2, showed that 10 bioactive phytochemical compounds were detected in both HME and HE. Each compound was characterized by its retention time (RT), molecular formula, molecular weight (MW), and concentration (area %).

Figure 2GC‐MS chromatogram of (a) HME and (b) HE.

**Figure:**
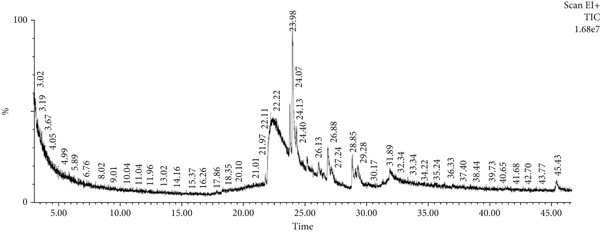
(a)

**Figure:**
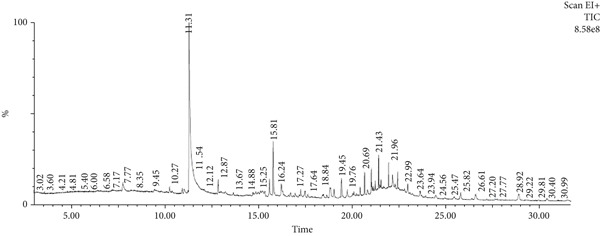
(b)

**Table 3:** Chemical compounds identified in HME and HE.

**No.**	**Compound name**	**Retention time**	**Area %**	**Molecular formula**	**Molecular weight (g/mol)**
GC‐MS analysis of HME					
1	Estragole	22.221	31.338	C_10_H_12_O	148.2
2	Methyl 2,4‐tridecadiynoate	22.714	18.029	C_14_H_20_O_2_	220.31
3	(6‐Hydroxymethyl‐2,3‐dimethylphenyl) methanol	23.232	8.534	C_10_H_14_O_2_	166.22
4	Beta‐Farnesene	23.781	5.643	C_15_H_24_	204.35
5	Alpha‐Bergamotene	23.985	15.154		
6	Androstane‐11,17‐dione, 3‐[(trimethylsilyl)oxy]‐, 17‐[O‐(phenylmethyl)oxime], (3à,5à)‐	24.312	2.841	C_29_H_43_NO_3_Si	481.7
7	Eicosapentaenoic acid	26.125	1.314	C_20_H_30_O_2_	302.5
8	Alpha‐Copaene	26.878	3.518	C_15_H_24_	204.35
9	Neophytadiene	28.852	3.469	C_20_H_38_	278.5
10	E‐6‐Octadecen‐1‐ol acetate	29.284	1.390	C_20_H_38_O_2_	310.5

GC‐MS analysis of HE					
11	6‐Methyl‐octadecane	3.024	13.270	C_19_H_40_	268.5
12	Decamethylcyclopentasiloxane	7.774	44.069	C_10_H_30_O_5_Si_5_	370.7
13	Benzoic acid, 4‐methyl‐2‐trimethylsilyloxy‐, trimethylsilyl ester			C_14_H_24_O_3_Si_2_	296.5
14	1‐Heptatriacotanol	9.088	5.841	C_37_H_76_O	537
15	2,7‐Dimethyl‐2,6‐octadien‐1‐ol	9.489	1.793	C_10_H_18_O	154.25
16	Stearic acid 2‐hydroxy‐1‐methylpropyl ester	9.816	1.062	C_22_H_44_O_3_	356.6
17	Estragole	11.315	17.714	C_10_H_12_O	148.2
18	Alpha‐Bergamotene	15.806	2.177	C_15_H_24_	204.3
19	Hexadecamethylcyclooctasiloxane	18.871	1.026	C_16_H_48_O_8_Si_8_	593.2
20	Tau‐Cadinol acetate	19.445	1.005	C_17_H_28_O_2_	264.4

### 3.4. TLC and HPTLC

TLC and HPTLC techniques were employed to separate and identify the diverse components within the extract. Vanillin–sulfuric acid reagent was utilized as a spraying agent to detect terpenoids, phenylpropane derivatives, phenols, and other similar compounds (Figure 3). The presence of multiple bands suggests that both extracts contain a diverse array of compounds, with different polarities, MWs, or chemical properties.

Figure 3Chromatograms of extract (a) TLC of HME, (b) TLC of HE, and (c) HPTLC analysis of both HE (left) and HME (right) after derivatization.

**Figure:**
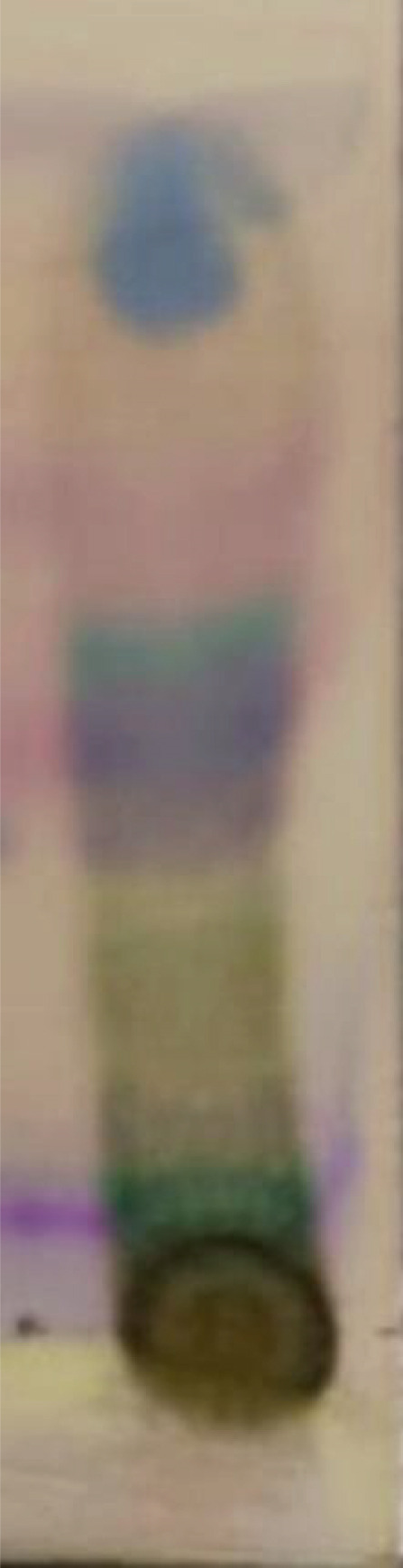
(a)

**Figure:**
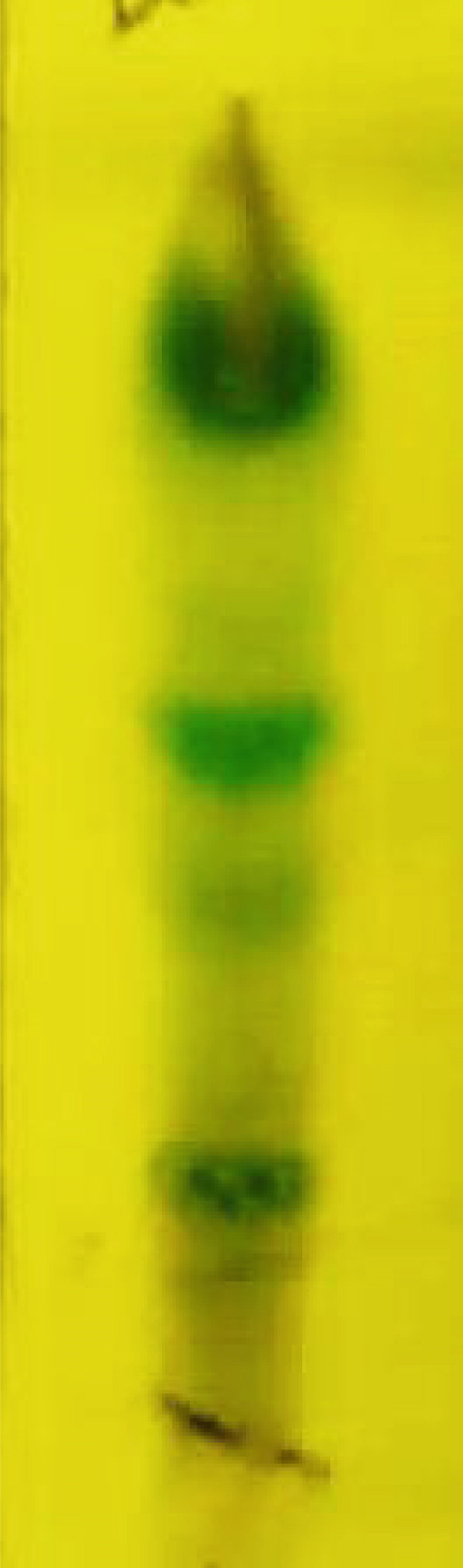
(b)

**Figure:**
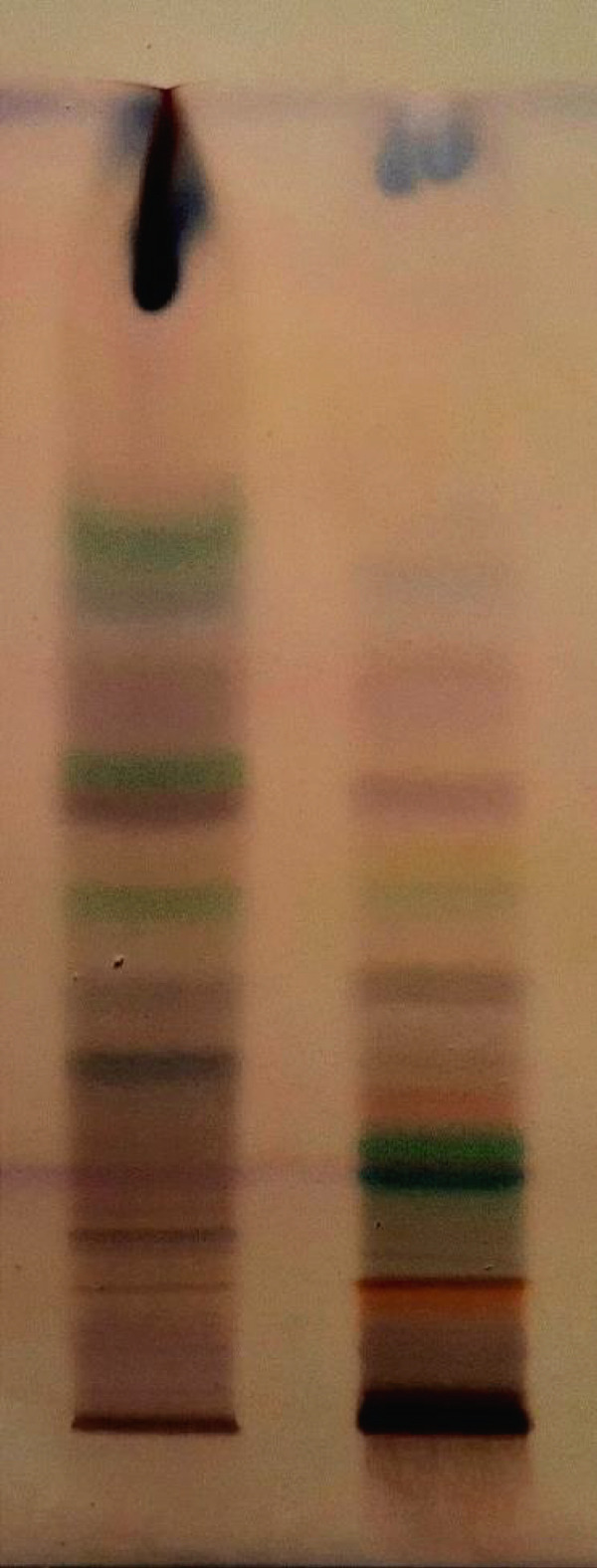
(c)

### 3.5. Determination of Total Phenolic, Flavonoid, and Tannin Contents

In the present study, HME contained a significantly higher quantity of phenols, flavonoids, and tannins compared to HE. Figure 4 illustrates the standard calibration curve showing linearity for gallic acid, quercetin, and tannic acid in the range of 10–50 *μ*g/mL, with a correlation coefficient (*R* ^2^) of 0.9978, 0.9915, and 0.9898, respectively.

Figure 4Standard calibration curve of (a) gallic acid, (b) quercetin, and (c) tannic acid. (d) Comparative analysis of total phenolic content (TPC), total flavonoid content (TFC), and total tannin content (TTC) in HME and HE.

**Figure:**
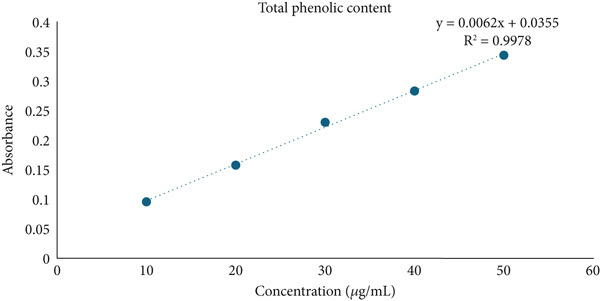
(a)

**Figure:**
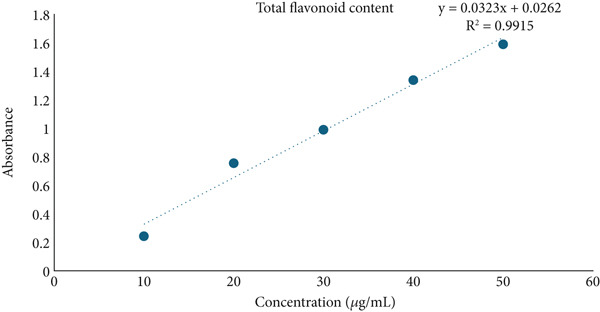
(b)

**Figure:**
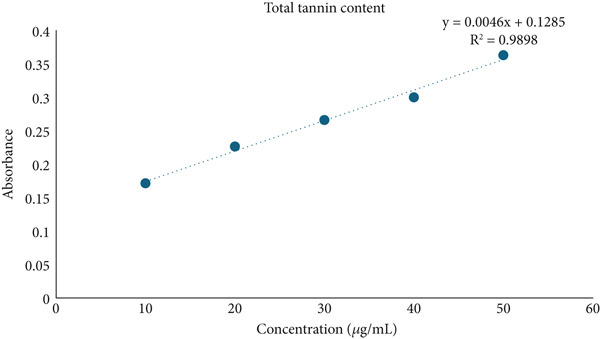
(c)

**Figure:**
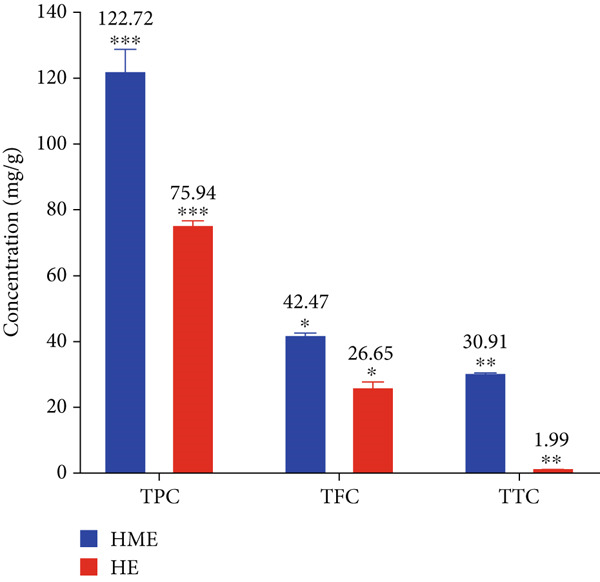
(d)

### 3.6. Antioxidant Activity of HME and HE

The antioxidant properties of HME and HE were assessed using DPPH, ABTS, NO, and SO assays. In a dose‐dependent manner, HME exhibited scavenging activities against DPPH, ABTS, NO, and SO radicals with IC_50_ values of 185.58 ± 2.17, 262.96 ± 12.94, 453.88 ± 6.47, and 651.09 ± 5.28 *μ*g/mL, respectively. Conversely, HE showed comparatively modest scavenging activity for these radicals, with IC_50_ values of 525.89 ± 12.07, 663.51 ± 12.07, 740.55 ± 7.87, and 844.27 ± 10.97 *μ*g/mL, respectively (Figure 5).

Figure 5Evaluation of antioxidant activity of HME and HE against ascorbic acid as standard. (a) DPPH, (b) ABTS, (c) nitric oxide, and (d) superoxide anion assay. (e) Comparative study of the IC_50_ (micrograms per milliliter) values of HME and HE for all assays. Statistical significance was determined as follows:  ^∗∗^ *p* < 0.01,  ^∗∗∗^ *p* < 0.001, and  ^∗∗∗∗^ *p* < 0.0001.

**Figure:**
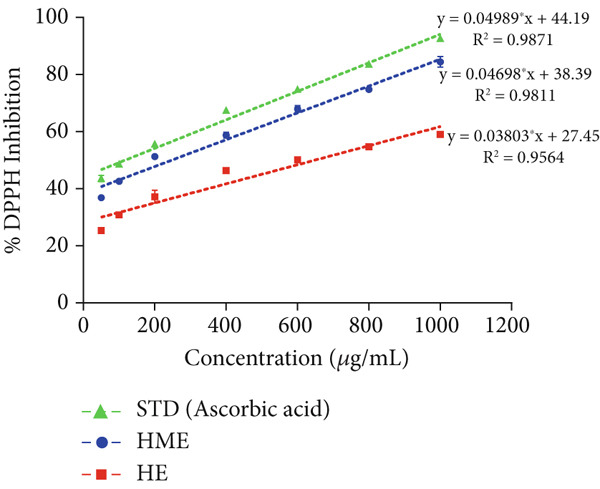
(a)

**Figure:**
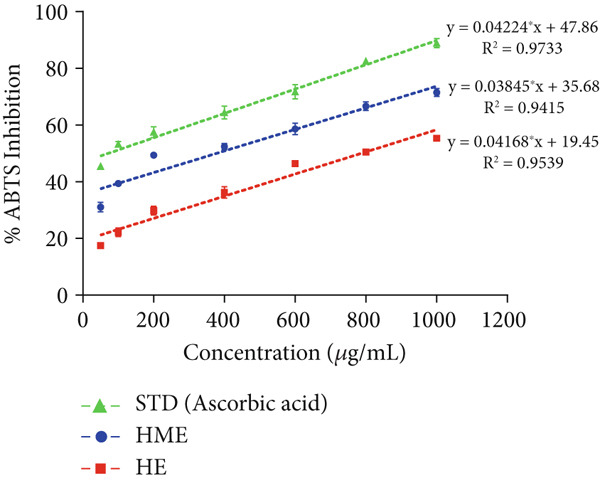
(b)

**Figure:**
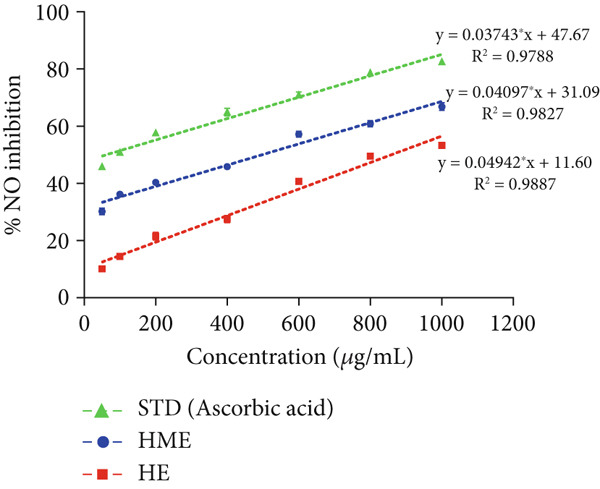
(c)

**Figure:**
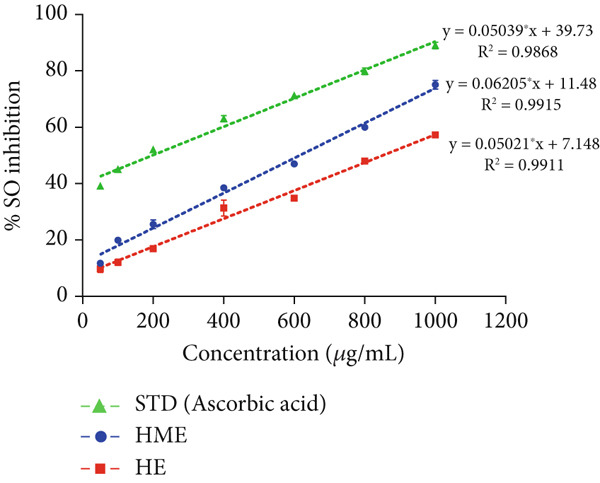
(d)

**Figure:**
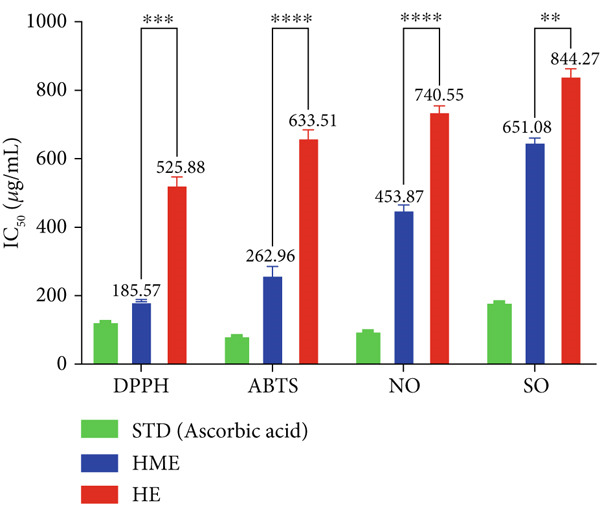
(e)

### 3.7. MTT Assay

Figure 6 illustrates the cytotoxic potential of both HME and HE extract on various cancerous cell lines. Both extracts exhibited cytotoxic effects on HCT 116, MCF‐7, and HeLa cells in a concentration‐dependent and time‐dependent manner. However, it was observed that HE demonstrated slightly higher cytotoxic effects compared to HME on all cancer cell lines. After 24 h of treatment, the IC_50_ values for cells treated with HME were 31.78 ± 0.48 (HCT 116), 53.28 ± 1.70 (MCF‐7), and 50.09 ± 1.1 *μ*g/mL (HeLa), while those treated with HE were 22.61 ± 0.77 (HCT 116), 37.24 ± 1.05 (MCF‐7), and 24.04 ± 0.8 *μ*g/mL (HeLa). Following 48 h of treatment, the IC_50_ values for cancer cells treated with HME decreased to 9.66 ± 0.35 (HCT 116), 43.52 ± 1.06 (MCF‐7), and 31.1 ± 1.4 *μ*g/mL (HeLa), while for HE‐treated cancer cells, they decreased to 6.27 ± 0.19 (HCT 116), 26.77 ± 0.21 (MCF‐7), and 12.6 ± 0.5 *μ*g/mL (HeLa).

Figure 6Cell viability (%) of HME and HE was assessed after 24 and 48 h on (a, d) HCT 116, (b, e) MCF‐7, and (c, f) HeLa cells. IC_50_ (micrograms per milliliter) values were compared for both extracts on (g) HCT 116, (h) MCF‐7, and (i) HeLa cells. Statistical significance was determined as follows:  ^∗∗^ *p* < 0.01,  ^∗∗∗^ *p* < 0.001, and  ^∗∗∗∗^ *p* < 0.0001.

**Figure:**
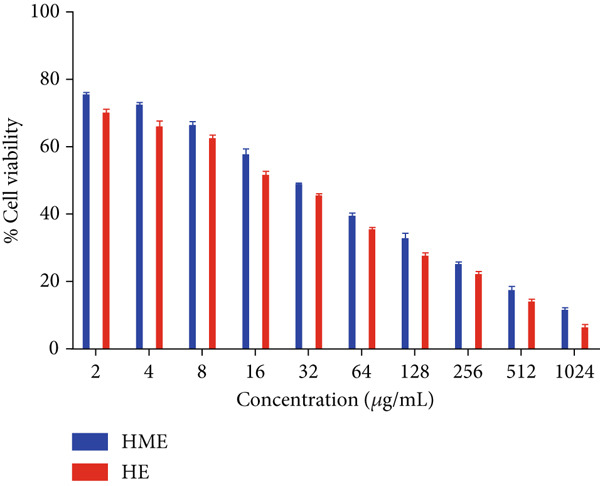
(a)

**Figure:**
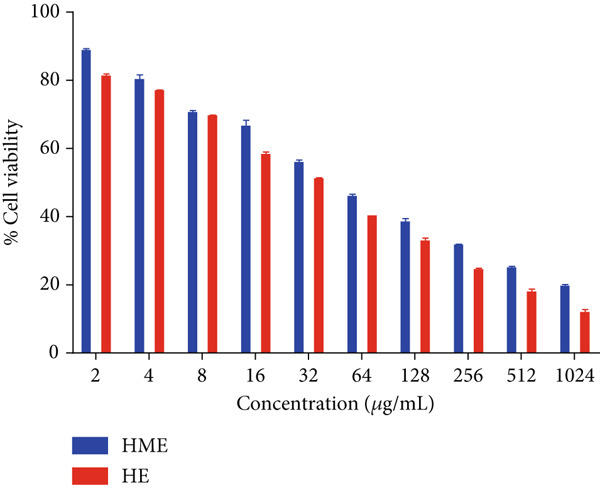
(b)

**Figure:**
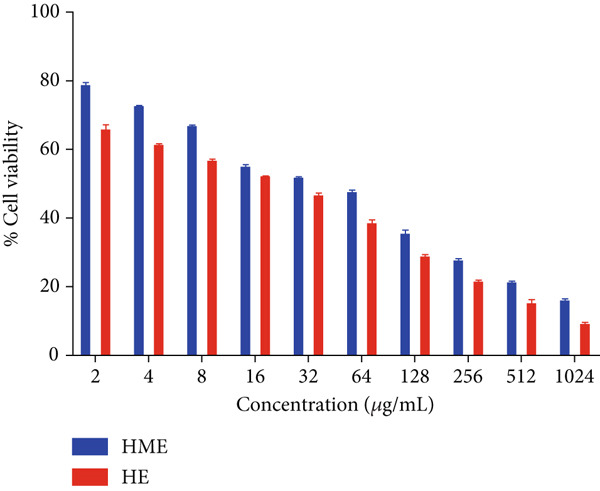
(c)

**Figure:**
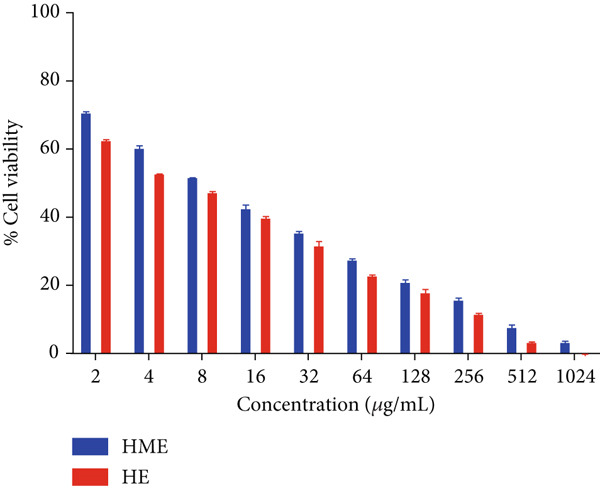
(d)

**Figure:**
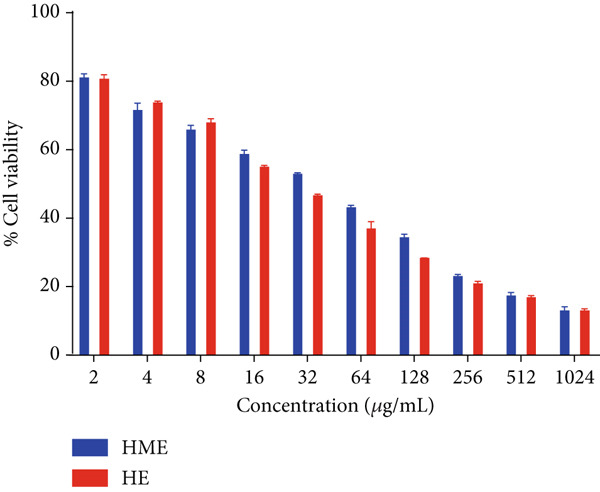
(e)

**Figure:**
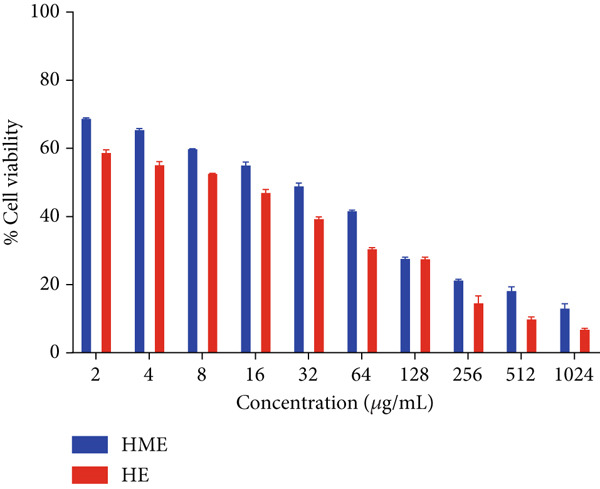
(f)

**Figure:**
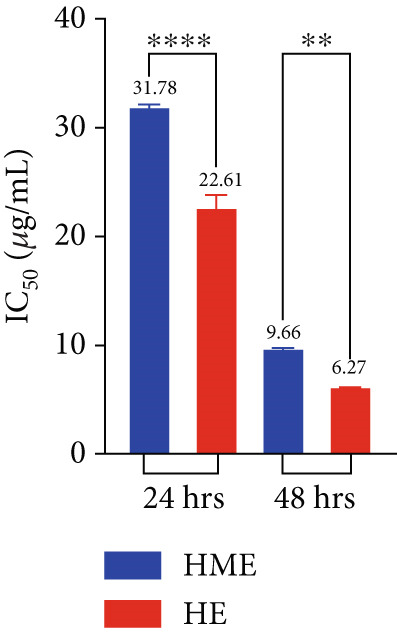
(g)

**Figure:**
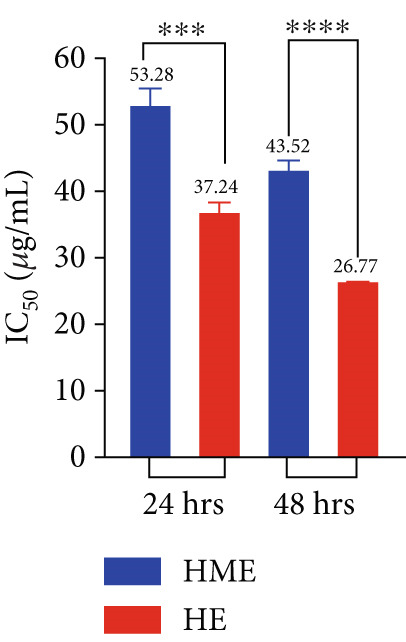
(h)

**Figure:**
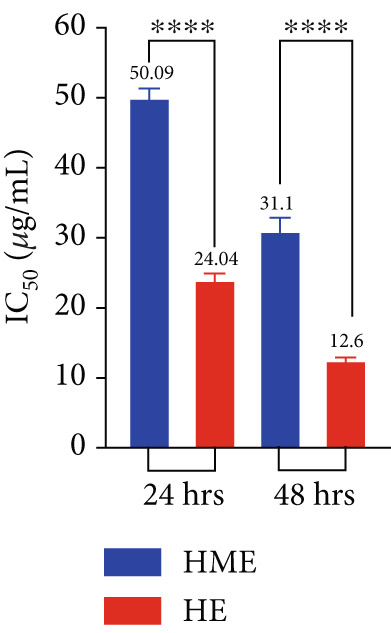
(i)

### 3.8. Toxicity Testing

#### 3.8.1. BSLA

**Figure 7:**
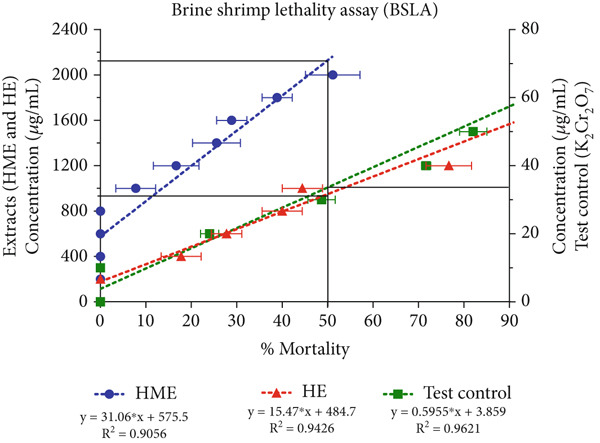
Brine shrimp lethality assay of HME, HE, and test control (K_2_Cr_2_O_7_). All results represent findings from three independent experiments.

Figure 7 illustrates that even at a concentration of 2000 *μ*g/mL, HME did not induce 50% mortality, indicating its relatively low toxicity. Conversely, HE demonstrated mild toxicity, with an LC_50_ value of 992 ± 88.34 *μ*g/mL, and its toxicity increased in a concentration‐dependent manner. These findings suggest that HME exhibits a higher safety margin at elevated concentrations, whereas HE possesses a lower toxicity threshold.

#### 3.8.2. Hemolysis Assay

**Figure 8:**
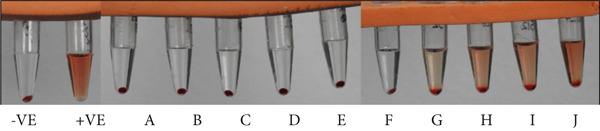
In vitro hemolysis assay of HME and HE: −VE = negative control, +VE = positive control, A to E = different concentrations of HME, and F to J = different concentrations of HE.

**Figure 9:**
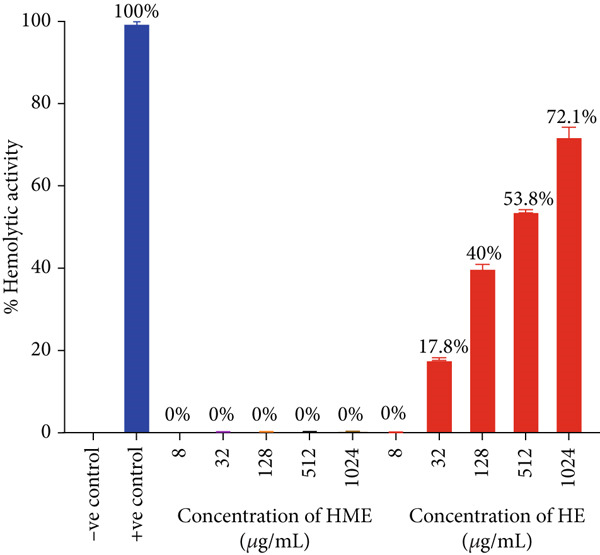
Comparison of hemolytic activity of HME and HE in reference to −VE control (PBS) and +VE control (Triton X‐100).

Figures 8 and 9 illustrate that HE exhibited a highly significant hemolytic effect on human red blood corpuscles erythrocytes, with 72.1% hemolysis observed at the highest concentration of 1024 *μ*g/mL. No hemolytic activity against human erythrocytes was observed at all concentrations of HME.

## 4. Discussion

Approximately 20% of identified plant species have undergone pharmaceutical investigation, significantly contributing to healthcare by addressing conditions such as cancer and other diseases [22]. Accurate plant authentication is crucial for ensuring the correct identification and sourcing of medicinal plants while preventing adulteration or misidentification [23]. This is achieved through scientific methods, including morphological examination, chemical profiling, and DNA barcoding [24]. In this study, BLASTn analysis of a 247 bp sequenced region (Table 1) revealed a 93.12% sequence identity with an *E*‐value of 3.00e − 102, indicating a highly significant match with minimal probability of random occurrence. Based on DNA barcoding, the analyzed leaf samples were conclusively identified as *Ocimum basilicum* L. FTIR spectroscopy analysis provided insights into the chemical composition and functional groups of the HME and HE extracts. The FTIR spectrum of HME exhibited diverse functional groups, including prominent O‐H stretching (2956.86–2852.05 cm^−1^), alkene vibrations (992.22–721.2 cm^−1^), and characteristic peaks corresponding to phenols, alcohols, isothiocyanates, imines, nitro compounds, sulfones, esters, and halogenated compounds. Conversely, HE exhibited fewer functional groups, primarily O‐H stretching (3245.38–2851.83 cm^−1^), with additional peaks corresponding to nitro, fluoro, and alkyl aryl ether functionalities. These spectral variations suggest distinct phytochemical compositions, which may influence the bioactivity of each extract. Furthermore, GC‐MS analysis of HME and HE identified a diverse range of bioactive phytochemicals with previously reported pharmacological potential. The compound estragole is known for its antioxidant [25], antimicrobial [26], anti‐inflammatory [27], and anticancer [28] properties. 1‐Heptatriacotanol has antimicrobial, anticonvulsant, antidepressant, and anti‐inflammatory effects [29]. Similarly, bergamotenes showed a wide range of biological activities, including antioxidants, anti‐inflammatory, immunosuppressive, cytotoxic, antimicrobial, glucose‐lowering, and insecticidal properties [30]. Tau‐Cadinol acetate displayed antiparasitic activity [31], while farnesene exhibited antibacterial [32], antifungal [33], free radical scavenging [34], and anticarcinogenic effects [35]. Eicosapentaenoic acid has been reported to ameliorate chronic kidney disease, lower cardiovascular risk, and reduce inflammation [36]. Additionally, it has been shown to induce apoptosis in in vitro human ovarian cancer cells [37]. Alpha‐Copaene containing essential oils have demonstrated anticarcinogenic, antioxidant, hepatoprotective, and anti‐inflammatory activities [38, 39], while neophytadiene exhibited anti‐inflammatory [40] and anticancer properties [41]. These findings suggest that *Ocimum basilicum* L. extracts possess a spectrum of bioactive compounds with potential therapeutic applications. Ahmed and coworkers in 2019 identified key constituents in *Ocimum basilicum* L. essential oils, including linalool, estragole, methyl cinnamate, eucalyptol, *α*‐bergamotene, eugenol, *γ*‐cadinene, germacrene D, and bicyclosesquiphellandrene. Among these, estragole and *α*‐bergamotene were also detected in both the HE and HME. Additionally, the HME contained related terpenoids such as *β*‐farnesene, *α*‐copaene, and neophytadiene, further supporting the similarity to the essential oil’s chemical profile and reflecting the plant’s characteristic phytochemical composition [42]. Further, to analyze the complex phytochemical composition of HME and HE, TLC and HPTLC were employed. The vanillin–sulfuric acid reagent, a widely used TLC stain, enabled the detection of phenolics, flavonoids, terpenoids, and other secondary metabolites. Quantification of total phenolic, flavonoid, and tannin contents in HME and HE revealed that phenolics were the most abundant, followed by flavonoids and tannins. HME exhibited significantly higher levels of these phytochemicals compared to HE. A study by Kaurinovic et al. [43] reported total phenolic and flavonoid contents of 11.8 ± 0.02 mg GAE/g and 26.42 ± 0.01 *μ*g RE/g in aqueous *Ocimum basilicum* L. extracts. Ahmed et al. [42] reported that the total phenolic content (TPC) of *Ocimum basilicum* L. essential oils ranged from 24.6 to 41.3 mg PE/g, while the ethanol extracts showed TPC values up to 82.45 mg PE/g. In contrast, this study recorded substantially higher concentrations, with HME containing 122.72 ± 4.02 mg GAE/g, 42.47 ± 0.59 mg QE/g, and 30.91 ± 0.31 mg TAE/g, while HE contained 75.94 ± 0.93 mg GAE/g, 26.65 ± 1.17 mg QE/g, and 1.99 ± 0.07 mg TAE/g. HME demonstrated significantly higher levels of phenolics (1.6‐fold, *p* < 0.001), flavonoids (1.5‐fold, *p* < 0.05), and tannins (16‐fold, *p* < 0.01) compared to HE, indicating that the selected extraction method effectively enhanced phytochemical yield. Extraction efficiency is significantly influenced by factors such as solvent type, temperature, and duration [44]. The findings of Djermane et al. [45] demonstrated that phenolic compounds are more effectively extracted using organic solvents of higher polarity and are consistent with the results of the present study. Given the strong correlation between phenolic and flavonoid contents of plant extracts and their antioxidant capacity, studies carried out by Koysu et al. [46], Zhao et al. [47], and Dede et al. [48] offer critical insight into the contribution of specific phenolic and flavonoid constituents to radical scavenging activity. This supports the current findings and emphasizes the importance of phenolic‐rich extracts in the development of natural antioxidant agents. The antioxidant potential of HME and HE was evaluated using DPPH, ABTS, NO, and SO assays. HME demonstrated significantly greater antioxidant activity across all assays. The most pronounced difference was observed in the DPPH assay, where HME’s radical scavenging ability was 2.8‐fold higher than HE (*p* < 0.001). In the ABTS, NO, and SO assays, HME exhibited antioxidant activity that was 2.5‐fold (*p* < 0.0001), 1.6‐fold (*p* < 0.0001), and 1.3‐fold (*p* < 0.01) higher than HE, respectively. These results suggest that the superior antioxidant activity of HME is directly linked to its higher concentrations of phenolics, flavonoids, and tannins. In comparison to the findings of Ahmed et al. [42], where ethanol extracts of *Ocimum basilicum* L. exhibited a DPPH IC_50_ value of 1.29 mg/mL and essential oils recorded IC_50_ values around 11.23 mg/mL, the hydromethanolic extract analyzed in the current study demonstrated greater antioxidant activity, with a DPPH IC_50_ of 185.58 *μ*g/mL (0.185 mg/mL). Additionally, the HE showed an IC_50_ of 525.89 *μ*g/mL (0.526 mg/mL), further indicating a comparatively higher free radical scavenging potential than the essential oil preparations. Previous studies by Gülçin et al. [49] and Nadeem et al. [50] similarly reported strong antioxidant potential in *Ocimum basilicum* L. extracts [49, 50]. Antioxidants play a crucial role in neutralizing free radicals, thereby mitigating oxidative stress–related cellular damage [51], which may contribute to anticancer activity through the inhibition of cancer cell proliferation or induction of apoptosis [52]. The cytotoxic potential of HME and HE was evaluated using the MTT assay against HCT 116, MCF‐7, and HeLa cancer cell lines. HE demonstrated significantly higher cytotoxic activity than HME against all three cell lines after both 24 and 48 h of incubation. The cytotoxic effect of HE was more pronounced after 48 h compared to 24 h. Specifically, HE’s activity after 24 h was 1.4 times greater (*p* < 0.0001) on HCT 116 cells, 1.4 times greater (*p* < 0.001) on MCF‐7 cells, and 2.1 times greater (*p* < 0.001) on HeLa cells. After 48 h, HE’s activity increased to 1.7 times greater (*p* < 0.01) on HCT 116 cells, 1.6 times greater (*p* < 0.0001) on MCF‐7 cells, and 2.4 times greater (*p* < 0.0001) on HeLa cells. The findings from multiple studies regarding the cytotoxic effects of *Ocimum basilicum* L. extracts on various cancer cell lines correlate well with the results of this study. Asl and coworkers in 2022 found significant cytotoxicity against colon cancer cell lines LS174T and COLO205 [53]. Torres and colleagues in 2018 reported up to 80% cytotoxic effect on MCF‐7 breast cancer cells [54]. Abd El‐Azim and coworkers in 2015 demonstrated similar cytotoxicity against colon carcinoma (HCT 116) and liver cell lines (HEPG2) [55]. The combined focus on pharmacology and toxicity in research on medicinal plants is crucial for advancing safe and effective treatments. It helps bridge traditional knowledge with modern scientific validation, ensuring that plant‐derived products can be used therapeutically with confidence in their efficacy and safety profiles [56]. To assess the safety profile of the extracts, brine shrimp lethality and hemolysis assays were conducted. The BSLA is an efficient toxicological screening tool for plant extracts [50]. According to Clarkson’s toxicity index [57, 58], substances with LC_50_ values < 100 *μ*g/mL are highly toxic, 100–500 *μ*g/mL are moderately toxic, and 500–1000 *μ*g/mL are low toxic, while Meyer’s index categorizes substances with LC_50_ < 1000 *μ*g/mL as toxic [20]. HME did not exhibit toxicity in brine shrimp nauplii, as 50% mortality was not observed even at 2000 *μ*g/mL. In contrast, HE exhibited mild toxicity, with an LC_50_ of 992 ± 88.34 *μ*g/mL, and toxicity increased with higher concentrations (Figure 7). These findings suggest that HME may be safer at higher concentrations. Antitumor compounds face challenges in drug development due to their cytotoxic effects on normal cells, like erythrocytes. The hemolysis assay is crucial for assessing plasma membrane integrity and the safety of injectable drugs, providing a sensitive indicator of potential damage to nontarget cells [59]. Excessive hemolysis may lead to anemia, cytotoxicity, and immune reactions, making its assessment essential for determining pharmacological suitability [60, 61]. HE exhibited a significant hemolytic effect (Figures 8 and 9), indicating potential erythrocyte damage that may limit its therapeutic application unless modifications mitigate its cytotoxicity. In contrast, HME demonstrated no hemolytic activity at any tested concentration, highlighting its biocompatibility and potential for pharmacological applications. The absence of hemolysis suggests that HME is a promising candidate for therapeutic formulations requiring minimal cytotoxicity. Combining metabolomic and pharmacognostic studies provided a comprehensive understanding of both the extracts of *Ocimum basilicum* L. leaves, offering valuable insights into their quality, safety, and medicinal properties. These results suggest that the phytochemicals in HME hold promise as therapeutic agents for cancer treatment. Future investigations can further elucidate the underlying mechanisms and optimize the application of these extracts in cancer treatment strategies.

## Conflicts of Interest

The authors declare no conflicts of interest.

## Funding

No funding was received for this manuscript.

## Data Availability

The authors have nothing to report.
